# Supplementary material for: Plasma Proteomic Variables Related to COVID-19 Severity: An Untargeted nLC-MS/MS Investigation
Source: Int J Mol Sci. 2023 Feb 10;24(4):3570. doi: 10.3390/ijms24043570 (PMC9962231; doi:10.3390/ijms24043570)
Supplement: Supplementary file 1 [file ijms-24-03570-s001.zip › SUPPLEMENTARY FIGURES AND TABLES PLUS CAPTIONS_revised.pdf]

SUPPLEMENTARY FIGURES AND TABLES

Table S3: ***Cohort description of patients during the acute phase - age & gender.*** This table summarises the demographic characteristics of the subset collected within 21 days from the first positive COVID-19 test (patients with negative test excluded): Age and Sex overall and stratified by the WHO Score, p-value (p) and the corresponding statistical tests are reported.

		**Overall**	**Mild**	**Severe**		
**Participants (n)**		19	11	8	**p**	**test**
AGE (median [IQR])		57.00 [43.00, 67.00]	47.00 [36.00, 57.00]	66.50 [63.25, 70.25]	0.039	Wilcoxon
SEX (%)	Female	10 (52.6)	7 (63.6)	3 (37.5)	0.370	Exact Fisher
	Male	9 (47.4)	4 (36.4)	5 (62.5)		

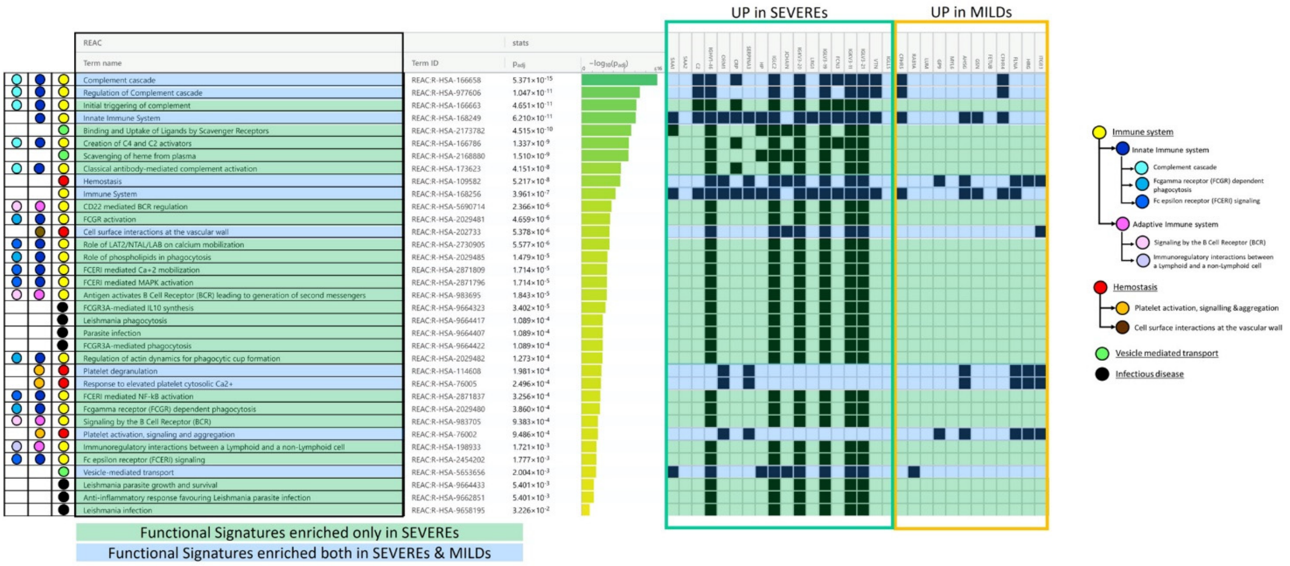

Figure S1: ***Reactome pathways enrichment.*** The image shows the reactome pathways enriched for the 29 proteins altered in MILDs and SEVEREs with g:Profiler [17]. In green are highlighted the functional signatures which are enriched only in Severe patients, while in blue those enriched both in Severe and Mild patients. Each REACTOME functionality has been put in relation with each deregulated protein (proteins up-expressed in SEVEREs are squared in aquamarine, while those up-expressed in MILDs are squared in orange), if one protein is involved in one function it is indicated by a black square. On the left, each functional signature has been categorised based on the hierarchical pathways clusterization reported in REACTOME database (the related legend box is reported on the right part of the image).

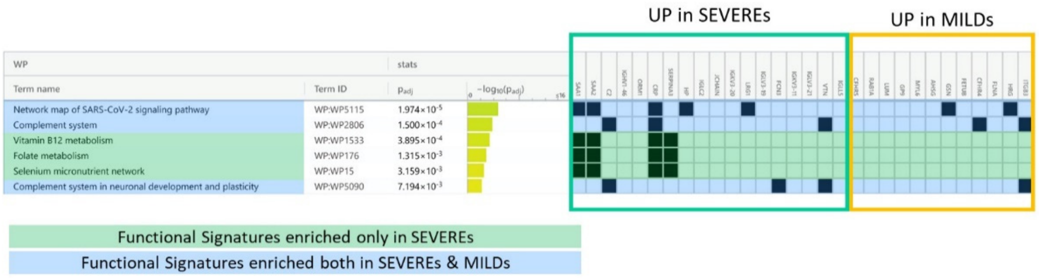

Figure S2: ***Wiki-pathways enrichment.*** The image shows the Wiki-pathways enriched for the 29 proteins altered in MILDs and SEVEREs with g:Profiler [17]. In green are highlighted the functional signatures which are enriched only in Severe patients while in blue those enriched both in Severe and Mild patients. Each wiki-pathway functionality has been put in relation with each deregulated protein (proteins up-expressed in SEVEREs are squared in aquamarine, while those up-expressed in MILDs are squared in orange), if one protein is involved in one function it is indicated by a black square.

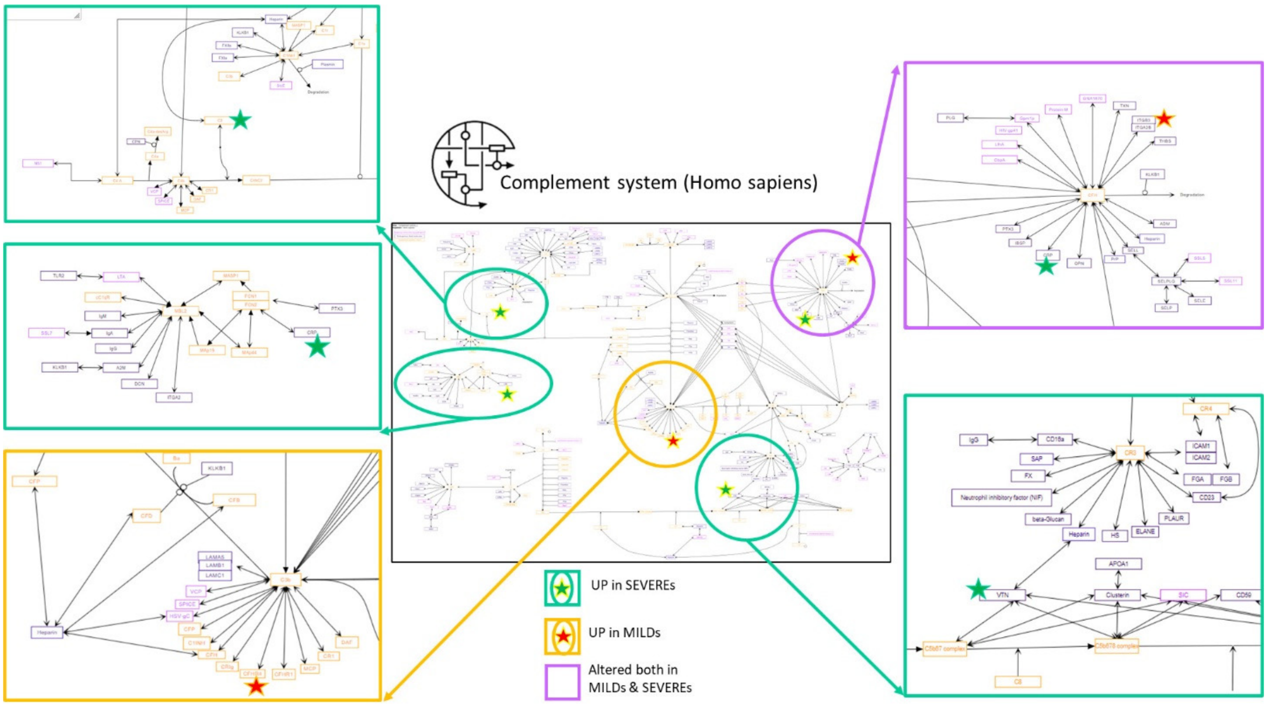

Figure S3: ***Wiki-pathways enrichment: complement system****.* Network map of the complement system in correlation with proteins altered in Mild and Severe SARS-CoV-2 patients based on WikiPathways graphics and g:profiler analysis (**Figure S2**) [17]. Nodes in aquamarine are deregulated in SEVEREs, nodes in orange are deregulated in MILDs, while nodes in purple are deregulated both in MILDs and SEVEREs. The green and red stars indicate the proteins which are up-expressed in Severe and Mild patients respectively.

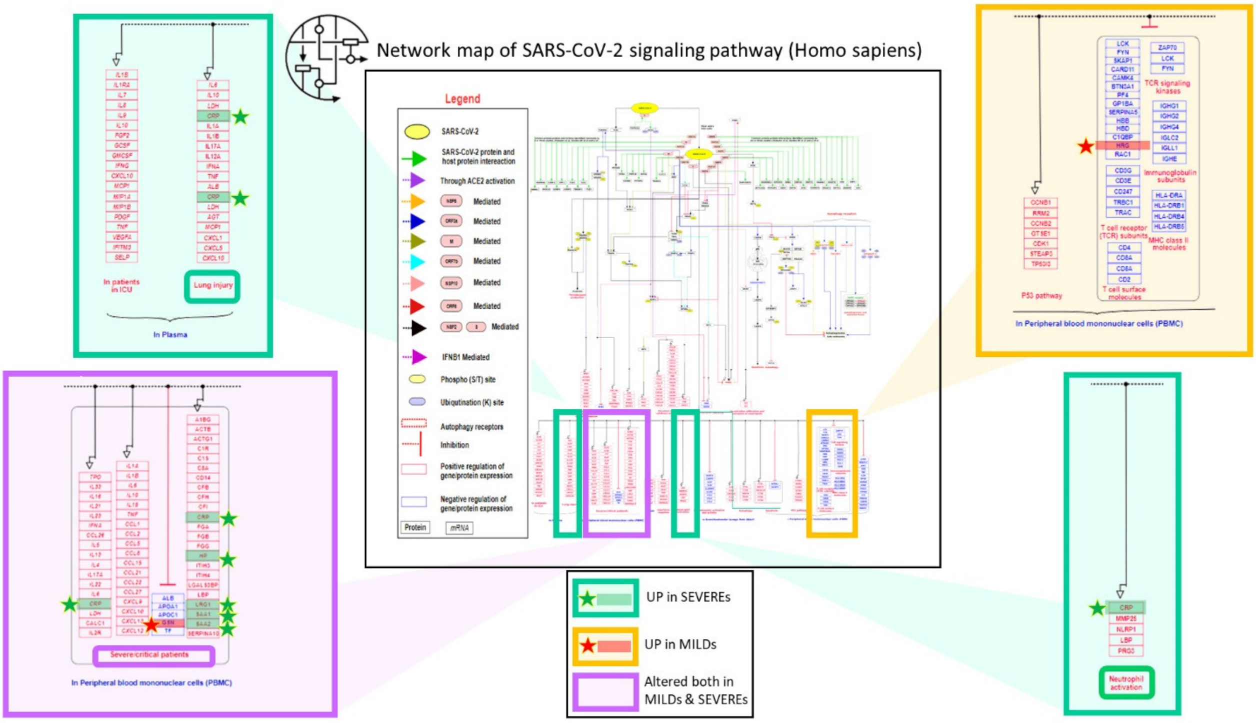

Figure S4: ***Wiki-pathways enrichment: network map of SARS-CoV-2 signalling pathway***. Network map of the SARS-CoV-2 signalling pathway in correlation with proteins altered in Mild and Severe SARS-CoV-2 patients based on WikiPathways graphics and g:profiler analysis (**Figure S2**) [17]. Nodes in aquamarine are deregulated in SEVEREs, nodes in orange are deregulated in MILDs, while nodes in purple are deregulated both in MILDs and SEVEREs. The green and red stars indicate the proteins which are up-expressed in Severe and Mild patients respectively.

Table S6: ***WHO score for COVID-19 disease severity***. This table correlates the gravity score assigned by the WHO with the patient state and with a more detailed description of the symptoms [8].

PATIENTS STATE	DESCRIPTION	SCORE
**Uninfected**	No clinical or virological evidence of infection	0
**Ambulatory**	**No limitation of activities**	**1**
	Limitation of activities	2
**Hospitalised - mild disease**	Hospitalised, no oxygen therapy	3
	**Oxygen by mask or nasal prongs**	**4**
**Hospitalised - severe disease**	**Non-invasive ventilation or high-flow oxygen**	**5**
	Intubation and mechanical ventilation	6
	Ventilation + additional organ support (pressors, renal replacement therapy (RRT), extracorporeal membrane oxygenation (ECMO))	7
